# Motherwort Injection for Preventing Postpartum Hemorrhage in Women with Vaginal Delivery: A Systematic Review and Meta-Analysis of Randomized Evidence

**DOI:** 10.1155/2019/1803876

**Published:** 2019-07-01

**Authors:** Jiajie Yu, Yujia Cai, Guanyue Su, Youping Li

**Affiliations:** ^1^Chinese Evidence-Based Medicine Center, West China Hospital, Sichuan University, Chengdu 610041, China; ^2^Editorial Office of the Chinese Journal of Evidence-Based Medicine, West China Hospital, Chengdu 610041, China; ^3^School of Preclinical and Forensic Medicine, Sichuan University, Chengdu 610041, China

## Abstract

**Background:**

Motherwort injection, a common traditional Chinese medicine, is widely used for the prevention of postpartum hemorrhage (PPH), which has been found to be potential benefit in clinical practice.

**Objectives:**

This study aimed to conduct a rigorous systematic review of randomized evidence to offer a comprehensive overview regarding the efficacy and safety of motherwort injection in maternal women with virginal delivery.

**Methods:**

We included all randomized controlled trials involving pregnant women in vaginal delivery comparing motherwort injection or combination of motherwort injection and oxytocin with oxytocin alone for preventing postpartum hemorrhage. Paired reviewers independently screened citations, assessed risk of bias, and extracted data. Random-effects model by Mantel-Haenszal method was applied to pool the data. Predefined subgroup analyses and sensitivity analyses were conducted to explore the heterogeneity and robustness of results. The GRADE approach was used to rate the quality of evidence.

**Main Results:**

37 randomized controlled trials involving 7887 participants were included, all of which were at moderate to high risk of bias. Meta-analyses of eight trials showed no significant difference in blood loss and PPH events between oxytocin versus motherwort injection (very low quality). However, pooling of 29 trials suggested a reduced risk of blood loss (within 2 hours: MD -55.06mL, 95% CI -84.06 to -26.06; within 24 hours: MD -85.57 mL, 95% CI -94.26 to -76.88, very low quality), PPH events (RR 0.29, 95% CI 0.21 to 0.39, low quality), and adverse events (Peto OR 0.53, 95% CI 0.40 to 0.70, very low quality) in participants treated with motherwort injection and oxytocin versus oxytocin alone.

**Conclusions:**

The current evidence supports the suggestion that the additional use of motherwort injection on oxytocin had a preferable outcome. However, given that the evidence is not definitive with low quality, further careful designed and conducted randomized controlled trials in larger population are warranted to conform the effects.

## 1. Introduction

Globally, approximately 830 women died every singer day due to complications during pregnancy or childbirth in 2015 (MMR was 216/1000000) [1]. Nearly 73% of all maternal deaths were due to direct obstetric causes such as abortion, embolism, hemorrhage, hypertension, and sepsis [2]. Hemorrhage was the leading direct cause of maternal deaths globally (27.1%), and more than two-thirds of hemorrhage deaths were postpartum hemorrhage (PPH) [3]. And almost all of these death (99%) occurred in low and middle-income countries [1]. 80% of PPH in pregnant women caused by uterine atony and most of these maternal deaths are preventable with necessary medication [4–6].

Uterotonic agents, including oxytocin, ergometrine, misoprostol, tranexamic acid, and carboprost, act on uterine muscles to induce uterine contraction and were initially introduced for prevention and treatment of PPH [7]. The first-line uterotonic agents which are recommended by World Health Organization and other international guidelines are oxytocin [8–12]. However, the need for cool storage and sterile equipment is the barriers to offer oxytocin production in resource-poor setting [13].

Motherwort injection extracted from motherwort (*Leonurus japonicus* Houtt), a common traditional Chinese herb for gynecologic disease in China for thousands years, is widely used for preventing PPH in China since 1972 [14]. The researches showed that motherwort injection worked on lower uterus without receptor saturation effect, which reduced the risk of adverse events caused by excessive use of oxytocin [15]. Moreover, motherwort injection is always worked by intramuscular when refrigeration and infusion are not readily available [16].

Given that the use of motherwort injection into routine practice is common in China, studies addressing the effects of motherwort injection are accumulating in the past years. Most of clinical trials and experience showed that prophylactic use of motherwort injection alone or combined with oxytocin was likely to have promise outcomes for preventing PPH after delivery [17, 18]. However, no well-designed and conducted systematic review that explores the efficacy and safety of motherwort injection alone or combined with oxytocin has been found now.

This study aimed to conduct a rigorous systematic review of randomized evidence to offer a comprehensive overview regarding the efficacy and safety of motherwort injection in maternal women with vaginal delivery.

## 2. Materials and Methods

We followed the reporting standards for systematic reviews and meta-analyses of randomized controlled trials according to PRISMA statements [19]. Ethical approval was not required for not involving human participants or human subjects' data.

### 2.1. Study Selection

We included randomized controlled trials if they recruited pregnant women anticipating a vaginal delivery; compared oxytocin with motherwort injection alone or combination of motherwort injection and oxytocin (the dose of oxytocin were comparable); and reported predefined outcomes including postpartum hemorrhage (estimated blood loss≥400mL within 2 hours or blood loss≥500mL within 24 hours), mean blood loss within 2 hours (mL), mean blood loss within 24 hours (mL), and adverse events.

Studies where participants received motherwort injection or oxytocin after blood loss more than 500ml or administrated contraction inhibitor medicine 48 hours prior to delivery were excluded.

### 2.2. Data Sources and Searches

We searched PubMed, EMbase, Cochrane Central Register of Controlled Trials (CENTRAL), Chinese database Sino-Med, Chinese National Knowledge Infrastructure Database (CNKI), VIP Chinese Science and Technique Journals Database, and WanFang database from inception to Dec 2017, updated to Dec 2018. Mesh and keyword search terms included were “postpartum hemorrhage”, “PPH”, “oxytocin”, “motherwort injection”, “Yimucao injection”, and search strategies of “randomized controlled trial” recommended by Cochrane Handbook [20]. We also searched ClinicalTrial.gov and Chinese Clinical Trial Register to identify additional eligible clinical trials. The reference lists of included studies were searched for additional eligible study. No restriction in language was applied.

### 2.3. Data Selection

Two reviewers (Su GY & Yu JJ) used predefined, pilot-tested forms to screen studies for eligibility, independently screened titles/abstracts, and full text of potential eligible articles. They independently assessed risk of bias and extracted data. Discrepancies were resolved through discussion, if necessary, arbitrated by a third reviewer (Li YP).

### 2.4. Risk of Bias Assessment

We assessed risk of bias of RCT using modified Cochrane Risk of Bias tool that include response options of “definitely or probably yes (assigned a low risk of bias)” or “definitely or probably no (assigned a high risk of bias)” [21–23]. The items included randomization sequence generation, allocation concealment, blinding of patients and personnel, or outcome assessors, infrequent missing outcome data, selective outcome reporting, and other sources of bias (industry funded).

### 2.5. Data Extraction

For all including trials, we collected information regarding study characteristic (sample size, publish year, author name, affiliation, and multicenter study), participants' characteristic (age, gestational week, and risk factors), interventions (dosage, timing, injection site, and duration of treatment), and outcomes (blood loss, the number of PPH, and adverse events).

### 2.6. Data Analysis and Rating Quality of Evidence

We conducted meta-analyses of all included trials. Heterogeneity among studies was assessed by Cochran's Q test and the I^2^ statistic. We applied the random-effects model using Mantel-Haenszel method to pool the data. We expressed dichotomous data as rate ratios (RRs) with 95% confidence intervals (CIs) and continuous data as mean differences (MDs) with 95% CIs. Considering the low event rate of adverse event, Peto's methods were also used [24]. If 10 or more studies would be included in the meta-analysis we will examine reporting biases by funnel plots and Egger's test [20].

We planned two subgroup hypotheses to explore source of heterogeneity: type of administration (immediate administration versus consecutive administration) and risk factors (women with high risk factor vs. women with no risk factor vs. unclear).

We conducted sensitivity analyses by using alternative effect measures (odds ratios vs. risk ratios), and statistical models pooling methods (Peto vs. Mantel-Hanszel method), regarding heterogeneity (random vs. fixed effects).

We also used the grading of recommendations assessment, development, and evaluation (GRADE) methodology to rate quality of the evidence and generate absolute estimated of effect for these outcomes [25].

## 3. Results

**Figure 1:**
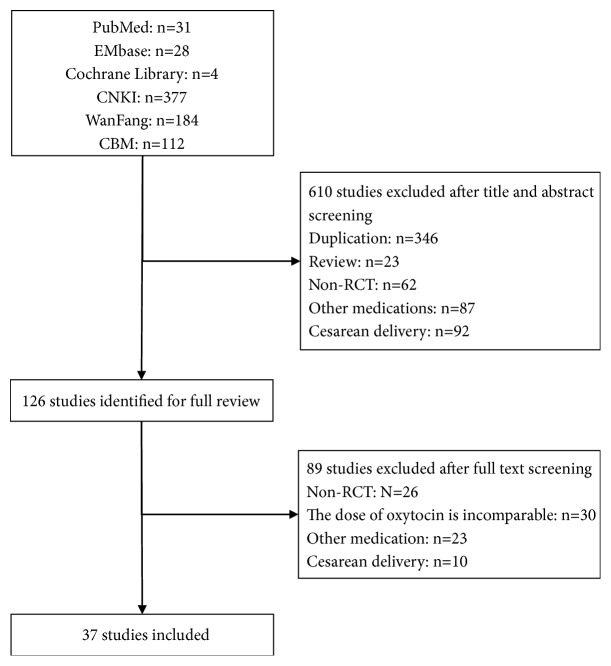
Study selection.

Of 736 unique reports, reviewers judged 126 as potential eligible after title and abstract screening. Of these, 37 trials involving 7887 women were proved eligible (Figure 1). These trials were all conducted in China between 2009 and 2018; the sample size ranged from 50 to 800; and the age of pregnant women ranged from 18 to 42. 28 trials assessed the combined treatment versus oxytocin alone, and eight compared motherwort injection with oxytocin (Table 1).

The risk of bias of including trials was moderate to high. Among these 37 trials, 13 (35.1%) adequately generated random sequence by random number table or computer; none of them clearly stated how to conceal the random sequence and blind the participants, doctors, or outcome assessors; 32 (86.5%) fails to complete the follow-up on outcome data; 20 were free of selective outcome reporting; and none of them reported the funding resource (Appendix 1).

All details of outcomes were found in Tables 2 and 3 and Appendix 3.

### 3.1. Motherwort Injection vs. Oxytocin

#### 3.1.1. Blood Loss within 2 Hours after Delivery (mL)

Eight RCTs involving 1793 participants reported blood loss within 2 hours after delivery. Substantial heterogeneity was present among those trials (I^2^=99%). The date from these trials showed no significant difference in blood loss within 2 hours after delivery between motherwort injections and oxytocin (MD: -21.78, 95%CI -52.43 to 9.88). The subgroup analysis by type of administration and risk factors showed difference in varied subgroups (interaction P<0.001).

#### 3.1.2. Blood Loss within 24 Hours after Delivery (mL)

Eight trials (n=1791) reported data on blood loss within 24 hours after delivery. Statistically significant difference was found in the pooling of data between two groups (MD -51.95, 95%CI -70.91 to -32.99, I^2^=92%). Subgroup analysis by type of risk factors showed similar findings (interaction P=0.18).

#### 3.1.3. Postpartum Hemorrhage

Four trials reported 59 PPH events occurring in 658 maternal women after delivery (raw event rate: 8.9%). Pooling data showed no significant difference in the risk of PPH between women receiving motherwort injection versus oxytocin (RR: 0.82, 95%CI 0.50 to 1.35, I^2^=0).

#### 3.1.4. Adverse Events

Of six trials, 3 (50%) reported that no adverse event occurred during the follow-up, and 3 other trials reported 152 adverse events among 867 women (raw event rate: 17.5%). Meta-analysis across trials reporting at least one adverse event showed a decrease in the risk of adverse events in women using motherwort injection versus oxytocin (Peto OR: 0.12, 95%CI 0.08 to 0.17, I^2^=57%)

### 3.2. Motherwort Injection Plus Oxytocin vs. Oxytocin

#### 3.2.1. Blood Loss within 2 Hours after Delivery (mL)

29 RCTs (n=6060) reported blood loss within 2 hours after delivery, heterogeneity among studies was high (I^2^=100%). Pooling data showed the reduction of blood loss within 2 hours was significant higher in combined group compared to oxytocin alone (MD: -55.06, 95%CI -84.06 to -26.06). The subgroup analysis by type of administration did not suggest apparent difference (interaction P=0.33). However, the subgroup analysis by risk factors suggested women in high risk factors had a higher blood loss reduction compared to other group (interaction P=0.007, high risk factors vs. no risk factor vs. unclear: -71.66 vs. -69.33 vs. -29.15).

#### 3.2.2. Blood Loss within 24 Hours after Delivery (mL)

27 trials, totaling 5710 women, reported data on blood loss within 24 hours after delivery. Pooling data also showed statistically significant difference between combined treatment versus control (MD: -85.57, 95%CI -94.26 to -76.88). The subgroup analysis by type of administration and risk factors showed similar findings in maternal women (administration, interaction P=0.21; risk factors, interaction P=0.63).

#### 3.2.3. Postpartum Hemorrhage

18 trials reported 229 PPH events occurring in 4767 women. The pooling data from those trials demonstrated the combination of motherwort injection and oxytocin was associated with lower risk of postpartum hemorrhage in women with vaginal delivery (RR 0.29, 95% CI 0.21 to 0.39, I^2^=0%). The subgroup analysis of our two prespecified hypotheses showed no significant difference in PPH events (administration, interaction P=0.62; risk factors, interaction P=0.97).

#### 3.2.4. Adverse Events

Of 21 trials, 7 (33.3%) reported that no adverse event occurred and 14 trials reported 228 adverse events in 2853 women during follow-up. The raw rate of adverse events was 5.4% in combined treatment group and 9.8% in oxytocin group (Peto OR 0.53, 95% CI 0.40 to 0.70, I^2^=54%). The subgroup analysis by type of administration showed that women with immediate administration may have a higher risk of adverse events compared to consecutive administration (Peto OR 0.74, 95% CI 0.54 to 1.03 versus Peto OR 0.19, 95% CI 0.11 to 0.33, interaction P<0.001).

For the comparisons between motherwort injection and oxytocin, considering the small limited number of trials, high risk of bias, wide confident intervals, substantial heterogeneity, and publication bias, the quality of evidence in all outcomes was very low (Appendix 2). With regard to the combined treatment group, postpartum hemorrhage outcome was rated as low because of high risk of bias and publication bias. The other three outcomes were all rated as very low for high risk of bias, heterogeneity, and publication bias (Appendix 2). The sensitivity analysis using alternative effect measures (relative risk vs. odds ratio), statistical models (Mantel-Haenszel vs. Peto), and considerations on heterogeneity (random effect vs. fixed effect) did not show important change in the pooled effects.

## 4. Discussion

Our study is the largest direct comparison meta-analyses involving 37 RCTs (n=7887) to evaluate the efficacy and safety of motherwort injection in maternal women with virginal delivery. Two comparisons were conducted in our study, we firstly compared the efficacy of motherwort injection alone with oxytocin, and no significant difference was found in blood loss volume after delivery and the rate of postpartum hemorrhage events.

The second clinical investigation compares the efficacy of additional use of motherwort injection on oxytocin versus oxytocin alone. This comparison demonstrated a significant reduction in the blood loss and the rate of PPH events after delivery with the additional use of motherwort injection on oxytocin. It is noteworthy that given the low quality and significant heterogeneity on blood loss, the interpretation about blood loss volume should be cautious.

The WHO recommended traditional medicines as more accessible, more affordable, and more acceptable than western medicines in some countries [26]. However, many western trained physicians held the critical view that traditional medicine is unscientific, unsupported by clinical trials, and some dangerous [27]. The greatest strength of motherwort injection is that the active ingredients extracted from motherwort could directly act on the uterine smooth muscle to facilitate uterine contraction and hemostasis and have an obvious dose-effect relationship [28, 29]. This means that the add-on therapy of motherwort injection could reduce the adverse events caused by the extensive use of oxytocin, such as hypertension, arrhythmia, and water retention. Two comparisons in our study showed the similar findings with previous studies. The adverse events were less common in maternal women with motherwort injection (whether used alone or additional) than oxytocin alone. The heterogeneity is also acceptable in these two comparisons. A postmarketing safety surveillance and reevaluation of motherwort injection in China conducting in 2015 showed that the incidence of adverse drug reactions (ADRs) was only 0.79‰ (8/10 094), the reported adverse events mainly included fever, chills, eyelid edema, pruritus, rash, nausea, and palpitation. All of these ADRs were mild in severity [30].

An issue not covered by this SR is that of cost, none of including trials provided information about cost. An unpublished study that compared the cost-effectiveness of motherwort injection for PPH prevention to oxytocin in 2402 Chinese maternal women suggested that the incremental cost of combination group compared with oxytocin group for one percentage of PPH risk was 94.11 RMB ($13.9).

One previous systematic review conducted in 2015 included 13 trials (n=2186) explored the effect of motherwort injection and oxytocin on the prevention of PPH [31]. Compared to this study, we included a large number of studies and conducted more thorough analyses. The additional trials provided more reliable estimate of effect. Moreover, we defined our potentially eligible trials as comparable dose of oxytocin between motherwort injection and oxytocin versus oxytocin alone. Consistent with our findings, they found similar risk of adverse events (RR=0.63, 95%CI 0.37-1.05) and significant decreased risk of postpartum hemorrhage (RR=0.30, 95%CI 0.19-0.47).

## 5. Strengths and Limitations

We conducted a comprehensive systematic review including all published RCTs with rigorous methods to evaluate the effect of motherwort injection for women with vaginal delivery. We conducted a limited number of preplanned subgroup analyses to explore the differences in outcomes. And we also used GRADE tool rate, the quality of evidence that insisted on confirming the reliability of results.

Our study also has a few important limitations. Firstly, the trials included suffered from important methodological limitations; the potential high risk of bias with small sample size that those trials poses has weakened our inference of the treatment effects. Secondly, although predefined subgroup analyses were considered in our study, substantial heterogeneity in blood loss was also reported. This was unsurprising given the differences in measurement of blood loss, experience of midwife, and setting. Thirdly, all trials we included were conducted in Chinese population, which limited generalizability of the findings. Fourthly, limited number of trials provided the details of participants and interventions, and we cannot make better suggestions about optimal administration of motherwort injection.

## 6. Conclusions

The current evidence, however, is not definitive, suggesting that the additional use of motherwort injection on oxytocin has profitable outcomes on the prevention of postpartum hemorrhage. Given the low quality of including trials, more careful designed and conducted clinical trials with more intervention details in larger population are warranted.

## Figures and Tables

**Table 1:** Characteristics of included studies.

Study	Intervention	No. of participants	Age (year)	Gestation (weeks)	Usage	Dosage	Risk factor
			Mean (SD)	Mean (SD)			
Chen GY, 2008	M	100	NR	NR	Consecutive	140mg	No
	O	100	NR	NR		70U	
Chen XQ, 2012	M	100	28.9(3.9)	38.7(1.3)	Consecutive	140mg	No
	O	100	29.1(4)	38.6(1.2)		70U	
Li W, 2014	M	40	27.2(1.2)	39.1(1.3)	Consecutive	120mg	Yes
	O	40	26.3(0.9)	37.5(0.8)		30U	
Liu WL, 2011	M	40	NR	NR	Consecutive	60mg	No
	O	39	NR	NR		30U	
Lin JH, 2009	M	150	NR	NR	Consecutive	100mg	No
	O	149	NR	NR		40U	
Lu LQ, 2011	M	300	NR	NR	Immediate	40mg	No
	O	300	NR	NR		20U	
Ren J, 2009	M	33	27.6(2.9)	39.3(1.2)	Consecutive	100mg	No
	O	34	28.9(3.2)	39.1(1.1)		40U	
Sun YW, 2012	M	100	28.6(2.9)	39.1(1.1)	Consecutive	200mg	NR
	O	100	28.3(2.5)	39.2(1.0)		20U	

Cheng L, 2013	M+O	400	NR	NR	Immediate	20U+40mg	No
	O	400	NR	NR		20U	
Dai YX, 2015	M+O	90	29.0(2.9)	39.2(1.2)	Immediate	20U+40mg	Yes
	O	90	29.0(3.0)	38.6(1.5)		20U	
Huang LR, 2011	M+O	50	26.0(1.1)	37.6(1.1)	Immediate	20U+20mg	No
	O	50	NR	NR		20U	
Li N, 2009	M+O	90	NR	NR	Immediate	20U+20mg	No
	O	90	NR	NR		20U	
Liu F, 2018	M+O	42	23.5(4.1)	NR	Immediate	30U+120mg	NR
	O	42	24.6(4.2)	NR		30U	
Liu LE, 2016	M+O	223	NR	NR	Immediate	20U+40mg	No
	O	224	NR	NR		20U	
Liu JM, 2017	M+O	67	29.4(4.2)	39(1.9)	Immediate	30U+120mg	NR
	O	67	30(4.1)	39.7(2.0)		30U	
Liu YN, 2018	M+O	49	25.2(2.1)	NR	Consecutive	30U+120mg	NR
	O	47	24.4(1.9)	NR		30U	
Lv LZ, 2011	M+O	100	27.0(2.5)	38.2(2.4)	Immediate	10U+20mg	Yes
	O	100	26.0(3.1)	38.7(2.1)		10U	
Ma DY, 2016	M+O	70	28.0(6.5)	NR	Consecutive	20U+80mg	Yes
	O	70	26.0(5.7)	NR		20U	
Shi H, 2015	M+O	50	NR	NR	Consecutive	70U+140mg	No
	O	50	NR	NR		70U	
Si RGL, 2016	M+O	190	22-37	NR	Immediate	10U+20mg	No
	O	190		NR		10U	
Sun X, 2018	M+O	45	26.7(2.2)	39.2(1.2)	Immediate	30U+120mg	NR
	O	45	26.6(2.4)	39.2(1.3)		30U	
Sun YW, 2015	M+O	127	25.0(2.8)	40.1(0.1)	Immediate	20U+40mg	No
	O	127	26.0(2.8)	40.2(0.1)		20U	
Wang L, 2008	M+O	200	NR	NR	Immediate	20U+40mg	NR
	O	200	NR	NR		20U	
Wang YH, 2015	M+O	50	26.0(3.6)	39.2(1.2)	Consecutive	20U+80mg	NR
	O	50	26.0(3.2)	38.9(1.1)		20U	
Wang YX, 2014	M+O	150	NR	NR	Immediate	20U+40mg	NR
	O	150	NR	NR		20U	
Wei YB, 2016	M+O	56	22-38	38-42	Immediate	20U+20mg	No
	O	56				20U	
Wu JJ, 2018	M+O	25	NR	NR	Consecutive	70U+140mg	Yes
	O	25	NR	NR		70U	
Wu N, 2016	M+O	45	26.0(3.5)	39.7(1.5)	Consecutive	20U+80mg	NR
	O	45	27.0(3.3)	39.4(1.3)		20U	
Xue QJ, 2018	M+O	152	28.6(2.3)	39.5(1.3)	Consecutive	60U+120mg	No
	O	152	28.5(2.2)	39.2(1.2)		80U	
Yang XF, 2018	M+O	100	26.5(1.7)	39.2(1.3)	Immediate	20U+40mg	No
	O	100	26.7(1.5)	39.1(1.4)		20U	
Yuan WJ, 2015	M+O	200			Consecutive	20U+80mg	No
	O	200				20U/40U	
Yue H, 2011	M+O	50	NR	NR	Consecutive	70U+140mg	Yes
	O	50	NR	NR		70U	
Zhang HH, 2014	M+O	100	26.0(1.2)	38.3(1.3)	Consecutive	70U+140mg	Yes
	O	100	26.0(1.2)	39.2(1.0)		70U	
Zhao XY, 2011	M+O	50	27.0(1.5)	39.0(1.3)	Immediate	20U+20mg	NR
	O	50	27.0(1.7)	39.2(1.4)		20U	
Zheng XH, 2012	M+O	60	28.0(1.6)	39.1(1.2)	Immediate	10U+20mg	NR
	O	60	28.0(1.7)	39.2(1.2)		10U	
Zhu WC, 2009	M+O	108	27.0(2.5)	38.7(2.1)	Immediate	20U+40mg	NR
	O	108	26.0(3.1)	38.2(2.4)		20U	

Wang P, 2012	M	95	20-34	37-44	Consecutive	100mg	No
	O	95				40U	
	M+O	95				40U+100mg	

**Table 2:** Comparison of motherwort injection vs. oxytocin.

Outcomes	N(n)	Effect estimate (95%CI)	I^2^	Interaction P
*Blood loss within 2 hours after delivery (mL)*				
Overall	8 (1793)	-21.18 (-52.43, 9.98)	99%	
Type of administration				
Immediate administration	1 (600)	-87.60 (-92.32, -82.88)	—	<0.001
Consecutive administration	7 (1193)	-12.95 (-29.59, 3.69)	88%	
Risk factor for PPH				
No risk factor	6 (1513)	-70.71 (-74.78, -66.64)	98%	<0.001
High risk factor	1 (80)	39.00 (9.86, 68.14)	—	
Unclear	1 (200)	-20.00 (-24.62, 15.38)	—	
*Blood loss within 24 hours after delivery (mL)*				
Overall	8 (1791)	-51.95 (-70.91, -32.99)	94%	
Type of administration				
Immediate administration	1 (600)	-89.70 (-93.84, -85.56)	—	<0.001
Consecutive administration	7 (1191)	-44.59 (-70.84, -18.35)	92%	
Risk factor for PPH				
No risk factor	6 (1511)	-80.63 (-84.42, -76.84)	96%	0.18
High risk factor	1 (80)	-57.00 (-88.64, -25.36)	—	
Unclear	1 (200)	-85.00 (-92.07, -77.93)	—	
*Postpartum hemorrhage*				
Overall	4 (658)	0.82 (0.50, 1.35)	0%	
Type of administration				
Consecutive administration	4 (658)	0.82 (0.50, 1.35)	0%	—
Risk factor for PPH				
No risk factor	3 (578)	0.73 (0.30, 1.78)	32%	0.78
High risk factor	1 (80)	0.88 (0.35, 2.18)	0%	
*Adverse events*				
Overall	6 (1529)	0.12 (0.08, 0.17)	57%	
Type of administration				
Immediate administration	1 (600)	0.10 (0.07, 0.15)	—	0.07
Consecutive administration	5 (929)	0.24 (0.10, 0.57)	0%	
Risk factor for PPH				
No risk factor	5 (1329)	0.04 (0.02, 0.08)	0%	0.01
Unclear	1 (200)	0.25 (0.07, 0.93)	—	

**Table 3:** Comparison of motherwort injection and oxytocin vs. oxytocin alone.

Outcomes	N(n)	Effect estimate (95%CI)	I^2^	Interaction P
*Blood loss within 2 hours after delivery (mL)*				
Overall	29 (6060)	-55.06 (-84.06, -26.06)	100%	
Type of administration				
Immediate administration	18 (4297)	-45.91 (-87.57, -4.25)	100%	0.33
Consecutive administration	11 (1763)	-69.93 (-93.59, -46.27)	99%	
Risk factor for PPH				
No risk factor	12 (3460)	-69.33 (-108.01, -30.64)	100%	0.007
High risk factor	6 (870)	-71.66 (-103.41, -39.92)	97%	
Unclear	11 (1730)	-29.15 (-36.81, -21.49)	98%	
*Blood loss within 24 hours after delivery (mL)*				
Overall	27 (5710)	-85.57 (-94.26, -76.88)	95%	
Type of administration				
Immediate administration	17 (4043)	-93.61 (-109.52, -77.69)	98%	0.21
Consecutive administration	10 (1667)	-80.91 (-92.61, -69.21)	88%	
Risk factor for PPH				
No risk factor	11 (3206)	-80.90 (-94.98, -66.82)	96%	0.63
High risk factor	6 (870)	-84.72 (-98.01, -71.43)	61%	
Unclear	10 (1634)	-91.82 (-109.22, -74.43)	96%	
*Postpartum hemorrhage*				
Overall	18 (4767)	0.29 (0.21, 0.39)	0%	
Type of administration				
Immediate administration	12 (3523)	0.30 (0.21, 0.42)	0%	0.62
Consecutive administration	6 (1244)	0.24 (0.12, 0.51)	0%	
Risk factor for PPH				
No risk factor	8 (2811)	0.29 (0.18, 0.44)	0%	0.97
High risk factor	5 (820)	0.27 (0.14, 0.51)	0%	
Unclear	5 (1136)	0.31 (0.16, 0.57)	0%	
*Adverse events*				
Overall	21 (4793)	0.53 (0.40, 0.70)	54%	
Type of administration				
Immediate administration	15 (3653)	0.74 (0.54, 1.03)	0	<0.001
Consecutive administration	6 (1140)	0.19 (0.11, 0.33)	35%	
Risk factor for PPH				
No risk factor	10 (2987)	0.60 (0.42, 0.85)	77%	0.88
High risk factor	3 (350)	0.66 (0.18, 2.34)	—	
Unclear	8 (1456)	0.51 (0.30, 0.87)	0%	

## Supplementary Materials

Supplementary Materials Appendix 1 provided the assessment outcomes of risk of bias in all including randomized controlled trials. The risk of bias of including trials was moderate to high. Among these 37 trials, 13 (35.1%) adequately generated random sequence by random number table or computer; none of them clearly stated how to conceal the random sequence and blind the participants, doctors, or outcome assessors; 32 (86.5%) fails to complete the follow-up on outcome data; 20 were free of selective outcome reporting; and none of them reported the funding resource. Appendix 2-a demonstrated the GRADE evidence profile of motherwort injection versus oxytocin. For the comparisons between motherwort injection and oxytocin, considering the small limited number of trials, high risk of bias, wide confident intervals, substantial heterogeneity, and publication bias, the quality of evidence in all outcomes was very low. Appendix 2-b demonstrated the GRADE evidence profile of motherwort injection plus oxytocin versus oxytocin. Postpartum hemorrhage outcome was rated as low because of high risk of bias and publication bias. The other three outcomes were all rated as very low for high risk of bias, heterogeneity, and publication bias. Appendix 3 provided the forest plots of all comparisons. Motherwort injection vs. oxytocin: blood loss within 2 hours after delivery (mL); blood loss within 24 hours after delivery (mL); postpartum hemorrhage and adverse events. Motherwort injection plus oxytocin vs. oxytocin: blood loss within 2 hours after delivery (mL); blood loss within 24 hours after delivery (mL); postpartum hemorrhage and adverse events.
