# Supplementary material for: Motherwort Injection for Preventing Postpartum Hemorrhage in Women with Vaginal Delivery: A Systematic Review and Meta-Analysis of Randomized Evidence
Source: Evid Based Complement Alternat Med. 2019 Jul 1;2019:1803876. doi: 10.1155/2019/1803876 (PMC6632498; doi:10.1155/2019/1803876)
Supplement: Supplementary Materials — Appendix 1 provided the assessment outcomes of risk of bias in all including randomized controlled trials. The risk of bias of including trials was moderate to high. Among these 37 trials, 13 (35.1%) adequately generated random sequence by random number table or computer; none of them clearly stated how to conceal the random sequence and blind the participants, doctors, or outcome assessors; 32 (86.5%) fails to complete the follow-up on outcome data; 20 were free of selective outcome reporting; and none of them reported the funding resource. Appendix 2-a demonstrated the GRADE evidence profile of motherwort injection versus oxytocin. For the comparisons between motherwort injection and oxytocin, considering the small limited number of trials, high risk of bias, wide confident intervals, substantial heterogeneity, and publication bias, the quality of evidence in all outcomes was very low. Appendix 2-b demonstrated the GRADE evidence profile of motherwort injection plus oxytocin versus oxytocin. Postpartum hemorrhage outcome was rated as low because of high risk of bias and publication bias. The other three outcomes were all rated as very low for high risk of bias, heterogeneity, and publication bias. Appendix 3 provided the forest plots of all comparisons. Motherwort injection vs. oxytocin: blood loss within 2 hours after delivery (mL); blood loss within 24 hours after delivery (mL); postpartum hemorrhage and adverse events. Motherwort injection plus oxytocin vs. oxytocin: blood loss within 2 hours after delivery (mL); blood loss within 24 hours after delivery (mL); postpartum hemorrhage and adverse events. [file 1803876.f1.docx]

Appendix 1 Risk of bias in included randomized controlled trials

| Author, year | Randomization sequence generation | Allocation concealment | Blinding of participants and personnel | Blinded assessment PPH events | infrequent missing outcome data | free of selective outcome reporting | Industry funded |
| --- | --- | --- | --- | --- | --- | --- | --- |
| Chen GY, 2008 | Unclear | High | High | High | Unclear | Unclear | Unclear |
| Chen XQ, 2012 | Unclear | High | High | High | High | Unclear | Unclear |
| Cheng L, 2013 | Unclear | High | High | High | Unclear | High | Unclear |
| Dai YX, 2015 | Unclear | High | High | High | Unclear | Low | Unclear |
| Huang LR, 2011 | Unclear | High | High | High | Unclear | Unclear | Unclear |
| Li N, 2014 | Unclear | High | High | High | Unclear | Unclear | Unclear |
| Li Wang, 2014 | Unclear | High | High | High | Unclear | High | Unclear |
| Lin JH, 2009 | Low | High | High | High | High | Low | Unclear |
| Liu F, 2018 | Low | High | High | High | Unclear | Low | Unclear |
| Liu JM, 2017 | Low | High | High | High | Unclear | Low | Unclear |
| Liu LE, 2016 | Unclear | High | High | High | Unclear | Low | Unclear |
| Liu WL, 2011 | Low | High | High | High | Unclear | Low | Unclear |
| Liu YN, 2018 | Unclear | High | High | High | High | Unclear | Unclear |
| Lu LQ, 2011 | Unclear | High | High | High | Unclear | Low | Unclear |
| Lv LZ, 2011 | Unclear | High | High | High | Unclear | Unclear | Unclear |
| Ma DY, 2016 | Unclear | High | High | High | Unclear | Low | Unclear |
| Ren J, 2009 | Unclear | High | High | High | High | Low | Unclear |
| Shi H, 2015 | Unclear | High | High | High | Unclear | Low | Unclear |
| Si RGL, 2016 | Unclear | High | High | High | Unclear | Low | Unclear |
| Sun X, 2018 | Low | High | High | High | Unclear | Low | Unclear |
| Sun YW, 2015 | Low | High | High | High | Unclear | Unclear | Unclear |
| Sun YW, 2012 | Unclear | High | High | High | Unclear | Low | Unclear |
| Wang L, 2008 | Unclear | High | High | High | Unclear | Unclear | Unclear |
| Wang P, 2012 | Unclear | High | High | High | Unclear | Low | Unclear |
| Wang YH, 2015 | Unclear | High | High | High | Unclear | Unclear | Unclear |
| Wang YX, 2014 | Unclear | High | High | High | Unclear | Low | Unclear |
| Wei YB, 2016 | Low | High | High | High | Unclear | Low | Unclear |
| Wu JJ, 2018 | Low | High | High | High | Unclear | Unclear | Unclear |
| Wu N, 2016 | Low | High | High | High | Unclear | Unclear | Unclear |
| Xue QJ, 2018 | Low | High | High | High | Unclear | Unclear | Unclear |
| Yang XF, 2018 | Low | High | High | High | Unclear | Unclear | Unclear |
| Yuan WJ, 2015 | Unclear | High | High | High | High | Low | Unclear |
| Yue H, 2011 | Low | High | High | High | Unclear | Low | Unclear |
| Zhang HH, 2014 | Unclear | High | High | High | Unclear | Low | Unclear |
| Zhao XY, 2011 | Unclear | High | High | High | Unclear | Low | Unclear |
| Zheng XH, 2012 | Unclear | High | High | High | Unclear | Unclear | Unclear |
| Zhu WC, 2009 | Low | High | High | High | Unclear | Unclear | Unclear |

Appendix 2-a GRADE evidence profile of motherwort injection versus oxytocin

| **Quality assessment** | | | | | | **Effect** | | **Quality** | **Importance** |
| --- | --- | --- | --- | --- | --- | --- | --- | --- | --- |
| **No of participants**  **(studies)** | **Risk of bias** | **Inconsistency** | **Indirectness** | **Imprecision** | **Publication bias** | **Relative (95% CI)** | **Absolute** |  |  |
| Blood loss within 2 hours after delivery (mL) | | | | | | | | | |
| 8 (1793) | Serious limitation, due to moderate to high risk of bias | Very serious limitation for high heterogeneity | No serious limitation | Serious limitation for wide confidence intervals | Serious limitation, due to poor methodological quality of small sample size studies | _ | MD 21.18 lower (52.34 lower to 9.98 higher) | ⊕⊝⊝⊝  Very low | Important |
| Blood loss within 24 hours after delivery (mL) | | | | | | | | | |
| 6 (1513) | Serious limitation due to moderate to high risk of bias | Very serious limitation for high heterogeneity | No serious limitation | Serious limitation for wide confidence intervals | Serious limitation, due to poor methodological quality of small sample size studies | _ | MD 51.95 lower (70.91 to 32.99 lower) | ⊕⊝⊝⊝  Very low | Important |
| Postpartum haemorrhage | | | | | | | | | |
| 4 (658) | Serious limitation due to moderate to high risk of bias | No serious limitation | No serious limitation | Serious limitation for wide confidence intervals | Serious limitation, due to poor methodological quality of small sample size studies | RR 0.81 (0.48 to 1.34) | 20 fewer per 1000 (from 54 fewer to 35 fewer) | ⊕⊕⊝⊝  Low | Critical |
| Adverse events | | | | | | | | | |
| 6 (1529) | Serious limitation due to moderate to high risk of bias | serious limitation for moderate heterogeneity | No serious limitation | No serious limitation | Serious limitation, due to poor methodological quality of small sample size studies | OR 0.12 (0.07 to 0.18) | 166 fewer per 1000 (from 155 fewer to 175 fewer) | ⊕⊝⊝⊝  Very low | Important |

Appendix 2-b GRADE evidence profile of combined treatment versus oxytocin

| **Quality assessment** | | | | | | **Effect** | | **Quality** | **Importance** |
| --- | --- | --- | --- | --- | --- | --- | --- | --- | --- |
| **No of participants**  **(studies)** | **Risk of bias** | **Inconsistency** | **Indirectness** | **Imprecision** | **Publication bias** | **Relative (95% CI)** | **Absolute** |  |  |
| Blood loss within 2 hours after delivery (mL) | | | | | | | | | |
| 29 (6060) | Serious limitation, due to moderate to high risk of bias | Very serious limitation for high heterogeneity | No serious limitation | No serious limitation | Serious limitation, due to poor methodological quality of small sample size studies | _ | MD 55.06 lower (84.06 to 26.06) | ⊕⊝⊝⊝  Very low | Important |
| Blood loss within 24 hours after delivery (mL) | | | | | | | | | |
| 27 (5710) | Serious limitation due to moderate to high risk of bias | Very serious limitation for high heterogeneity | No serious limitation | No serious limitation | Serious limitation, due to poor methodological quality of small sample size studies | _ | MD 85.57 lower (94.26 to 76.88 lower) | ⊕⊝⊝⊝  Very low | Important |
| Postpartum haemorrhage | | | | | | | | | |
| 18 (4767) | Serious limitation due to moderate to high risk of bias | No serious limitation | No serious limitation | No serious limitation | Serious limitation, due to poor methodological quality of small sample size studies | RR 0.28 (0.21 to 0.37) | 55 fewer per 1000 (from 48 fewer to 61 fewer) | ⊕⊕⊝⊝  Low | Critical |
| Adverse events | | | | | | | | | |
| 14 (2853) | Serious limitation due to moderate to high risk of bias | serious limitation for moderate heterogeneity | No serious limitation | No serious limitation | Serious limitation, due to poor methodological quality of small sample size studies | OR 0.53 (0.4 to 0.7) | 27 fewer per 1000 (from 17 fewer to 34 fewer) | ⊕⊝⊝⊝  Very low | Important |

Appendix 3 Forest plots of comparisons

Appendix fig 1 Motherwort injection vs oxytocin: Blood loss within 2 hours after delivery (mL)

Appendix fig 2 Motherwort injection vs oxytocin-subgroup analyses: Blood loss within 2 hours after delivery (mL): administration.

Appendix fig 3 Motherwort injection vs oxytocin: Blood loss within 24 hours after delivery (mL)

Appendix fig 4 Motherwort injection vs oxytocin-subgroup analyses: blood loss within 24 hours after delivery (mL): administration.

Appendix fig 5 Motherwort injection vs oxytocin: postpartum hemorrhage.

Appendix fig 6 Motherwort injection vs oxytocin-subgroup analyses: postpartum hemorrhage: administration

Appendix fig 7 Motherwort injection vs oxytocin: adverse events.

Appendix fig 8 Motherwort injection vs oxytocin-subgroup analyses: adverse events: administration.

Appendix fig 9 Motherwort injection + oxytocin vs oxytocin: blood loss within 2 hours after delivery (mL).

Appendix fig 10 Motherwort injection + oxytocin vs oxytocin - subgroup analyses: blood loss within 2 hours after delivery (mL): immediate vs consecutive administration

Appendix fig 11 Motherwort injection + oxytocin vs oxytocin - subgroup analyses: blood loss within 2 hours after delivery (mL): risk factor

Appendix fig 12 Motherwort injection + oxytocin vs oxytocin: blood loss within 24 hours after delivery (mL).

Appendix fig 13 Motherwort injection + oxytocin vs oxytocin - subgroup analyses: blood loss within 24 hours after delivery (mL): immediate vs consecutive administration.

Appendix fig 14 Motherwort injection + oxytocin vs oxytocin - subgroup analyses: blood loss within 24 hours after delivery (mL): risk factor.

Appendix fig 15 Motherwort injection + oxytocin vs oxytocin: postpartum hemorrhage

Appendix fig 16 Motherwort injection + oxytocin vs oxytocin - subgroup analyses: postpartum hemorrhage: immediate vs consecutive administration.

Appendix fig 17 Motherwort injection + oxytocin vs oxytocin - subgroup analyses: postpartum hemorrhage: risk factor.

Appendix fig 18 Motherwort injection + oxytocin vs oxytocin: adverse events.

Appendix fig 19 Motherwort injection + oxytocin vs oxytocin - subgroup analyses: adverse events: immediate vs consecutive administration.

Appendix fig 20 Motherwort injection + oxytocin vs oxytocin - subgroup analyses: adverse events: risk factor.
